# Prevalence of Genetically Complex *Leishmania* Strains With Hybrid and Mito-Nuclear Discordance

**DOI:** 10.3389/fcimb.2021.625001

**Published:** 2021-02-24

**Authors:** Hirotomo Kato, Abraham G. Cáceres, Eduardo A. Gomez, Ahmed Tabbabi, Daiki Mizushima, Daisuke S. Yamamoto, Yoshihisa Hashiguchi

**Affiliations:** ^1^ Division of Medical Zoology, Department of Infection and Immunity, Jichi Medical University, Tochigi, Japan; ^2^ Sección de Entomología, Instituto de Medicina Tropical “Daniel A. Carrión” y Departamento Académico de Microbiología Médica, Facultad de Medicina Humana, Universidad Nacional Mayor de San Marcos, Lima, Peru; ^3^ Laboratorio de Entomología, Instituto Nacional de Salud, Lima, Peru; ^4^ Departamento de Parasitología y Medicina Tropical, Facultad de Ciencias Médicas, Universidad Católica de Santiago de Guayaquil, Guayaquil, Ecuador; ^5^ Department of Parasitology, Kochi Medical School, Kochi University, Kochi, Japan

**Keywords:** *Leishmania*, hybrid, mito-nuclear discordance, genetic exchange, Ecuador, Peru

## Abstract

Approximately 20 *Leishmania* species are known to cause cutaneous, mucocutaneous, and visceral disorders in humans. Identification of the causative species in infected individuals is important for appropriate treatment and a favorable prognosis because infecting species are known to be the major determinant of clinical manifestations and may affect treatments for leishmaniasis. Although *Leishmania* species have been conventionally identified by multilocus enzyme electrophoresis, genetic analysis targeting kinetoplast and nuclear DNA (kDNA and nDNA, respectively) is now widely used for this purpose. Recently, we conducted countrywide epidemiological studies of leishmaniasis in Ecuador and Peru to reveal prevalent species using PCR-RFLP targeting nDNA, and identified unknown hybrid parasites in these countries together with species reported previously. Furthermore, comparative analyses of kDNA and nDNA revealed the distribution of parasites with mismatches between these genes, representing the first report of mito-nuclear discordance in protozoa. The prevalence of an unexpectedly high rate (~10%) of genetically complex strains including hybrid strains, in conjunction with the observation of mito-nuclear discordance, suggests that genetic exchange may occur more frequently than previously thought in natural *Leishmania* populations. Hybrid *Leishmania* strains resulting from genetic exchanges are suggested to cause more severe clinical symptoms when compared with parental strains, and to have increased transmissibility by vectors of the parental parasite species. Therefore, it is important to clarify how such genetic exchange influences disease progression and transmissibility by sand flies in nature. In addition, our aim was to identify where and how the genetic exchange resulting in the formation of hybrid and mito-nuclear discordance occurs.

## Introduction

Human leishmaniasis is caused by approximately 20 species of the genus *Leishmania* belonging to the subgenera *Leishmania (Leishmania), Leishmania (Viannia)*, and *Leishmania (Mundinia)* (Paranaiba et al., 2017; Ruiz-Postigo et al., 2020). The clinical presentation is varied, ranging from a localized cutaneous lesion to a potentially fatal visceral disorder, and the infecting parasite species is the major determinant of the outcome (Ruiz-Postigo et al., 2020). Importantly, several *L. braziliensis* complex species, such as *Leishmania (Viannia) braziliensis* and *L. (V.) guyanensis*, are associated with a risk of metastasizing destructive mucosal lesions after healing of the primary cutaneous lesion (Ruiz-Postigo et al., 2020). In addition, for cutaneous leishmaniasis, variability in disease severity and susceptibility to treatment may be as sociated with the infecting parasite species, although the characteristic cutaneous lesions caused by each infecting species have yet to be determined. Therefore, identification of the causative *Leishmania* species is important for appropriate treatment and a favorable prognosis.

*Leishmania* species have been classified by multilocus enzyme electrophoresis (MLEE) as the reference protocol (Rioux et al., 1990; Cupolillo et al., 1994). This method requires parasite isolation in culture, which is time-consuming and associated with risks of contamination with bacteria and fungi on sample collection, and interfusion of other cultures during long-term cultivation. Recently, the application of molecular biological techniques using samples directly obtained from patients’ lesions has facilitated rapid and efficient identification of the parasite species. Kinetoplast DNA (kDNA) is a unique mitochondrial structure found in trypanosomatid parasites, containing 20–50 copies of maxicircle DNA and approximately 10,000 copies of minicircle DNA (Simpson, 1986). Because of the multicopy property, kDNA is widely used as a target for detection and identification of *Leishmania* species. Although minicircle DNA is more sensitive for detection, it is heterogeneous in sequence. Therefore, maxicircle genes, such as cytochrome *b* (*cyt* b), cytochrome *c* oxidase subunits, and NADH dehydrogenase subunits, are preferentially used as targets for species identification; *cyt* b gene sequence analysis is widely used and accepted as a reliable marker for this purpose (Luyo-Acero et al., 2004; Asato et al., 2009; Kato et al., 2010; Kato et al., 2011; Leelayoova et al., 2013; Kato et al., 2016a; Kato et al., 2019a). Similarly, among nuclear DNA (nDNA) targets, internal transcribed spacer (ITS) regions of ribosomal RNA and heat shock protein 70 (*hsp70*) are commonly used for species identification, due to their sensitivity for detection of interspecific sequence divergence (da Silva et al., 2010; Talmi-Frank et al., 2010; de Almeida et al., 2011; Fraga et al., 2012; Montalvo et al., 2012). Generally, genetic analysis of a single target is considered acceptable for reliable identification at the species level; however, analysis of multiple targets may increase accuracy. Furthermore, analysis of single targets may not detect strains that are the product of recombination between different species.

Recently, countrywide surveillances were performed in Ecuador and Peru using *cyt* b gene analysis, and geographic distributions of *Leishmania* species were identified (Kato et al., 2010; Kato et al., 2016a; Kato et al., 2019a). Furthermore, comparative analyses of kDNA and nDNA revealed the prevalence of genetically complex *Leishmania* including hybrids and strains with mismatches between these genes, known as mito-nuclear discordance, at an unexpectedly high rate (~10%) in these countries (Kato et al., 2019b; Tabbabi et al., 2020). In this review, we describe the genetic complexity of *Leishmania* strains found in Ecuador and Peru that showed hybrid and mito-nuclear discordance characteristics, and discuss where and how such genetic exchange occurs, and its influence on disease severity and expansion of potential vector species.

## Leishmaniasis in Ecuador and Perú, and Identification of Causative Species Based on Cytochrome B Gene Analysis

**Figure 1:**
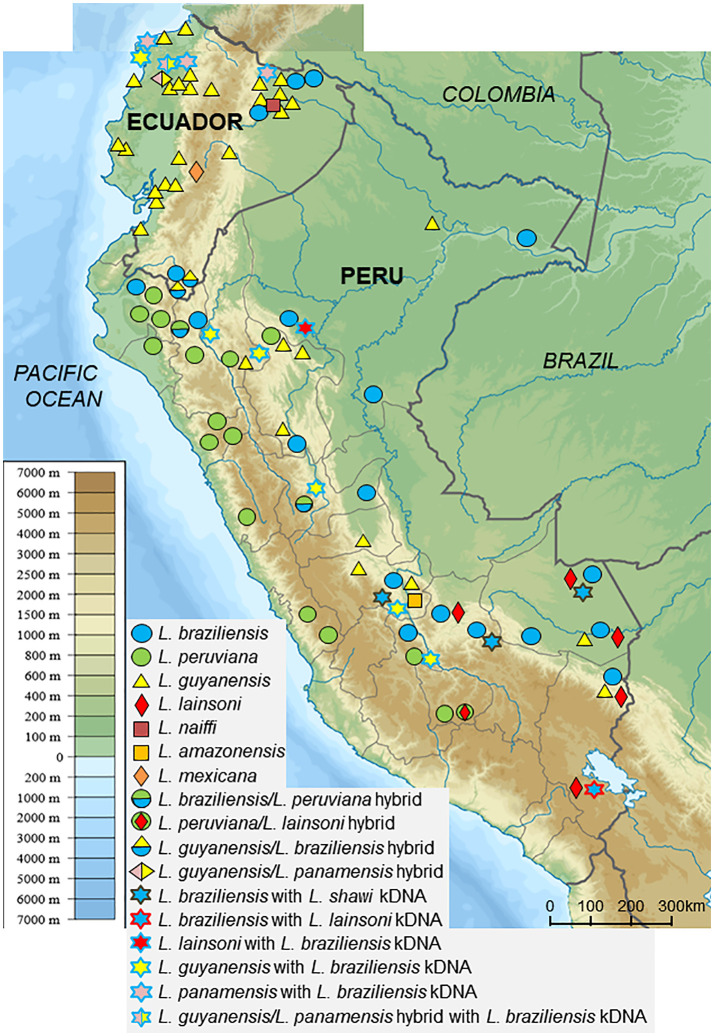
Geographic distribution of *Leishmania* species in Ecuador and Peru. *Leishmania* species in clinical samples were identified by sequence analysis of kinetoplast DNA and PCR-RFLP and sequence analyses of nuclear DNA. (Adapted from maps available at https://commons.wikimedia.org/wiki/File:Peru_physical_map.svg and https://commons.wikimedia.org/wiki/File:Ecuador_relief_location_map.svg).

Ecuador is a relatively small country located on the Equator in northwestern South America. The country includes four ecological regions, each with a unique biodiversity and ecosystem: the Pacific coast subtropical areas, Andean highlands, Amazonian rainforest, and Galapagos Islands. Leishmaniasis is endemic in the first three regions (Hashiguchi et al., 2017). Up to the present, eight *Leishmania* species: *L. (V.) guyanensis, L. (V.) panamensis, L. (V.) braziliensis, L. (V.) naiffi, L. (V.) lainsoni, L. (L.) mexicana, L. (L.) amazonensis*, and *L. (L.) major*-like, have been recorded as responsible for cutaneous leishmaniasis (CL) and mucocutaneous leishmaniasis (MCL) (Kato et al., 2016a; Hashiguchi et al., 2017; Kato et al., 2019b) (**Figure 1**). On the Pacific coast, *L. (V.) guyanensis* is the dominant causative agent, and infections by *L. (V.) panamensis* and *L. (V.) braziliensis* also have been reported. In addition, the distribution of *L. (L.) amazonensis* has been recorded in certain areas, although infection by it has not been reported recently (Kato et al., 2016a; Hashiguchi et al., 2017). In Amazonian areas, CL and MCL caused by *L. (V.) guyanensis* and *L. (V.) braziliensis* have been widely recorded, and CL caused by *L. (V.) naiffi* and *L. (V.) lainsoni* was recently reported in several areas (Kato et al., 2013; Kato et al., 2016a; Kato et al., 2016b). In the Andean highlands, areas endemic for CL are limited to the mid-southwestern part of Ecuador, and *L. (L.) mexicana* is currently the major causative species, whereas infection by *L. (L.) major*-like was reported previously (Kato et al., 2016a; Hashiguchi et al., 2017; Hashiguchi et al., 2018) (**Figure 1**). In addition, a hybrid of *L. (V.) guyanensis* and *L. (V.) braziliensis* was recorded in southern parts (Bañuls et al., 1997). The observed variety in species and hybrids that cause CL in this relatively small country may reflect the extensive ecological and biological diversities, including among sand fly vectors and reservoir animals. In Ecuador, CL is the most frequently observed form of leishmaniasis, most commonly presenting as an ulcer, followed by popular, nodular, and atypical forms, including diffuse and disseminated lesions, and recidiva cutis (Hashiguchi et al., 2016; Hashiguchi et al., 2017). The characteristic presenting symptoms have not been defined for all infecting species, however, diffuse and disseminated forms of leishmaniasis are caused by *L. (L.) mexicana* and *L. (V.) guyanensis*, respectively (Hashiguchi et al., 2017).

Peru, a larger country located to the south of Ecuador, similarly includes Pacific coast, Andean highland, and Amazonian rainforest regions. Peru is one of the most highly endemic countries for CL, which occurs throughout the country from highlands to lowlands. In contrast, MCL is endemic to Amazonian areas (Kato et al., 2010; Kato et al., 2019a). Six *Leishmania* species and several hybrids have been recorded as responsible for leishmaniasis (Kato et al., 2010; Kato et al., 2019a; Tabbabi et al., 2020) (**Figure 1**). Of these, the main causative agents are *L. (V.) peruviana*, *L. (V.) braziliensis*, and *L. (V.) guyanensis*, mainly circulating in the Andean highlands, tropical rainforest, and northern to central rainforest areas, respectively (Kato et al., 2010; Kato et al., 2019a) (**Figure 1**). In addition to the three dominant species, *L. (V.) lainsoni* and *L. (L.) amazonensis* have been reported to be cause disease in lower rainforest areas, and *L. (V.) shawi* was recently identified as a rare and sporadic species responsible for CL based on *cyt* b gene sequence analysis (Kato et al., 2010; Kato et al., 2019a) (**Figure 1**). Furthermore, a hybrid of *L. (V.) braziliensis* and *L. (V.) peruviana* first recorded in 1995 in a central area, was recently reported in northern Peru (Dujardin et al., 1995; Koarashi et al., 2016; Kato et al., 2019a) (**Figure 1**). Unlike Ecuador, CL is highly endemic throughout Andean areas in Peru. Additionally, the cutaneous lesions of patients in the Peruvian Andes were commonly larger and more severe when compared with those of patients in the Ecuadorian Andes (Hashiguchi et al., 2018).

## Comparative Nuclear and Kinetoplast DNA Analyses Reveal Genetically Complex *Leishmania* Strains With Hybrid and Mito-Nuclear Discordance

Sequence analysis targeting kDNA and nDNA is a powerful and reliable tool for the identification of infecting *Leishmania* species; however, it requires costly reagents and equipment. Therefore, cost-effective alternatives are preferable for more practical use applicable in less-equipped laboratories/countries. Of these, PCR-restriction fragment length polymorphism (RFLP) analysis is a promising candidate. In addition, PCR-RFLP allows analysis of heterozygous multicopy regions. For this purpose, nDNA is considered to be a more suitable target than kDNA, due to the potential effect of polymorphisms in the kDNA sequences of both minicircle and maxicircle genes on restriction fragment patterns. To date, ITS regions of ribosomal RNA and the *hsp70* gene have been widely used as targets due to their sensitivity, specificity, and reliability (Garcia et al., 2004; Rotureau et al., 2006; Spanakos et al., 2008; Fraga et al., 2012; Khanra et al., 2012; Fraga et al., 2013; Mouttaki et al., 2014). In addition, PCR-RFLP analyses targeting leishmanial mannose phosphate isomerase (*mpi*) and 6-phosphogluconate dehydrogenase (*6pgd*) genes, both of which have been used for multilocus sequence typing (MLST), were recently established, and their reliability for species identification was confirmed (Kato et al., 2019b).

**Figure 2:**
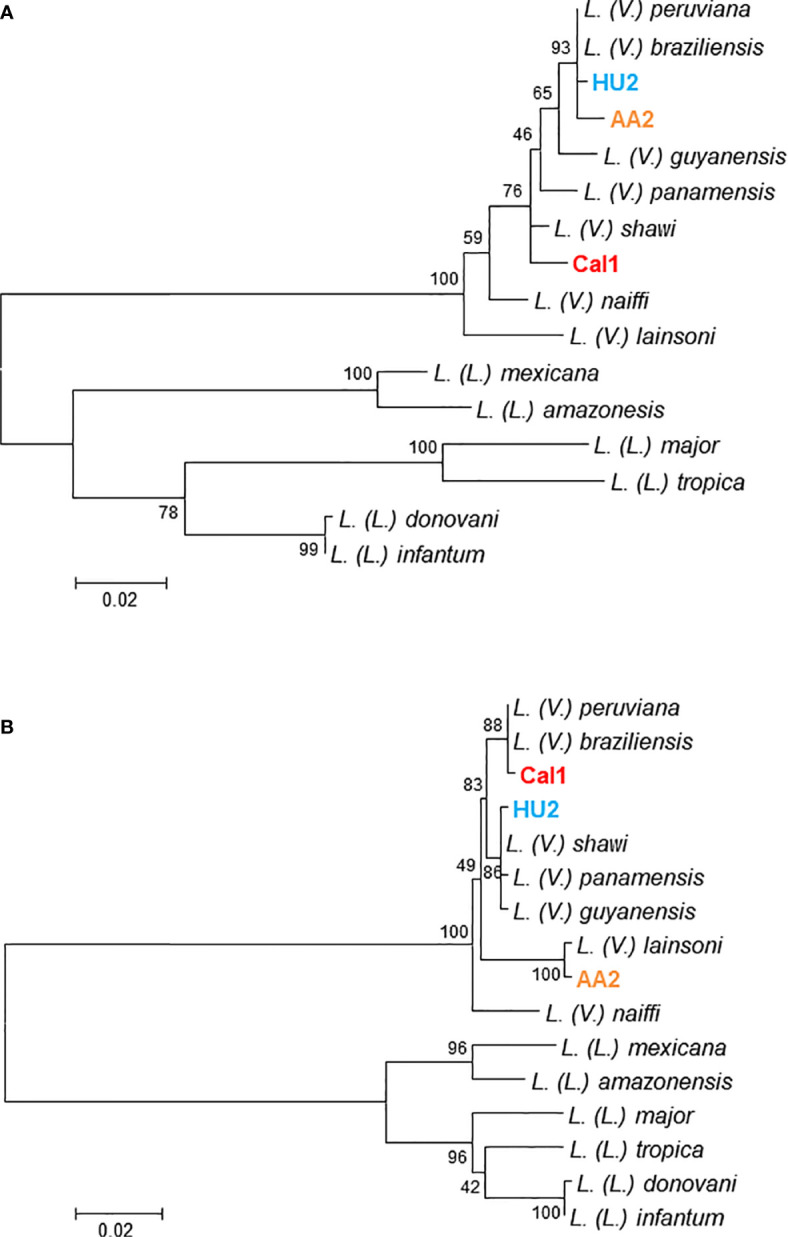
Discordance between cytochrome *b* and mannose phosphate isomerase gene sequences in clinical samples. Leishmanial cytochrome *b* **(A)** and mannose phosphate isomerase **(B)** genes were determined from clinical samples of patients with cutaneous leishmaniasis (HU2, AA2, and Cal1), and phylogenetic analyses were performed by the maximum likelihood method together with those sequences from 13 *Leishmania* species. The scale bar represents 0.02% divergence. Bootstrap values are shown above or below branches. Parasites were identified as *Leishmania (Viannia) guyanensis* with *L. (V.) braziliensis* kDNA, *L. (V.) lainsoni* with *L. (V.) braziliensis* kDNA, and *L. (V.) shawi* with *L. (V.) braziliensis* kDNA in clinical samples, HU2, AA2, and Cal1, respectively.

In recent studies, PCR-RFLP analyses of nDNA were applied to 92 and 134 clinical samples from Ecuador and Peru, respectively, and the results were compared with those obtained by kDNA sequence analyses (Kato et al., 2019b; Tabbabi et al., 2020). Interestingly, results that were consistent between the two analyses were obtained only for about 90% of samples each, from Ecuador and Peru (90.2 and 87.3%, respectively). On the other hand, five Ecuadorian samples showed hybrid patterns by PCR-RFLP, and were identified as hybrid strains of *L. (V.) guyanensis*/*L. (V.) braziliensis* and *L. (V.) guyanensis/L. (V.) panamensis* (Kato et al., 2019b). Similarly, six Peruvian samples showing hybrid RFLP patterns were identified as hybrids of *L. (V.) braziliensis*/*L. (V.) peruviana* and *L. (V.) peruviana*/*L. (V.) lainsoni* (Tabbabi et al., 2020) (**Figure 1**). Furthermore, these studies unexpectedly identified strains showing incompatibility between kDNA and nDNA, known as mito-nuclear discordance, which had not been reported previously in protozoa, in five Ecuadorian (5.4%) and eleven Peruvian (8.2%) samples (Kato et al., 2019b; Tabbabi et al., 2020). These samples were identified as hybrids of *L. (V.) guyanensis*/*L. (V.) panamensis* with *L. (V.) braziliensis* kDNA, *L. (V.) guyanensis* with *L. (V.) braziliensis* kDNA, and *L. (V.) panamensis* with *L. (V.) braziliensis* kDNA in Ecuador, and of *L. (V.) guyanensis* with *L. (V.) braziliensis* kDNA, *L. (V.) braziliensis* with *L. (V.) shawi* kDNA, *L. (V.) braziliensis* with *L. (V.) lainsoni* kDNA, and *L. (V.) lainsoni* with *L. (V.) braziliensis* kDNA, in Peru. Interestingly, strains with mito-nuclear discordance were detected from geographically separate areas, rather than from delimited areas in both countries (**Figures 1** and **2**). The distribution of unexpectedly high rates of hybrid or mito-nuclear discordance strains in both Ecuador and Peru indicates that the genetic structure of *Leishmania* is more complex than expected. In addition, these results suggest that interspecific genetic exchange occurs at a certain frequency in nature. It is important to note that all strains with mito-nuclear discordance are associated with *L. (V.) braziliensis*, suggesting that the species may have characteristics promoting genetic exchange with other species.

## Where and How Does Genetic Exchange Occur?

Hybrids of *Leishmania* species such as *L. (V.) braziliensis/L. (V.) guyanensis*, *L. (L.) infantum/L. (L.) major*, and *L. (L.) donovani/L. (L.) aethiopica* have been reported in other countries (Delgado et al., 1997; Ravel et al., 2006; Odiwuor et al., 2011). Recently, genome-scale analyses provided evidence of meiotic-like recombination between *Leishmania* species, resulting in full-genome hybrids (Van den Broeck et al., 2020). Interestingly, this study also showed that the mitochondrial genome of hybrid strains consisted of homogeneous uniparental maxicircles, whereas minicircles originated from both parental species (Van den Broeck et al., 2020).

The mechanisms of genetic exchange in *Leishmania* resulting in the formation of hybrid and mito-nuclear discordance, and where and how they occur, are still unclear. In Peru, the natural hybridization between *L. (V.) braziliensis* and *L. (V.) peruviana* is hypothesized to be associated with a massive migration of people and animals between highland and lowland areas, due to the deterioration and recovery of the political and security situation (Kato et al., 2016c; Van den Broeck et al., 2020). A resulting increased risk for infection by multiple *Leishmania* species in humans and animals, is thought to give rise to the emergence and establishment of hybrid strains. A hybrid of *Leishmania* can be generated experimentally in sand fly vectors by co-infecting them with two different strains of the same *Leishmania* species (Akopyants et al., 2009; Sadlova et al., 2011; Calvo-Álvarez et al., 2014), and *in vitro* by co-culture of different strains of *L. (L.) tropica* promastigotes, a stage in the sand fly vector lifecycle (Louradour et al., 2020). In addition, direct evidence of sexual recombination in natural populations was provided by whole genome sequencing of *Leishmania* isolated from sand flies (Rogers et al., 2014). The midgut adhesion molecule of sand flies is believed to be a major determinant of parasite-vector specificity by supporting species-specific parasite attachment and their growth (Kamhawi et al., 2004). Therefore, interspecific hybrid formation is considered to occur within sand flies if the two parasite species share the midgut molecule for their attachment, which may be possible between closely-related species such as *L. (V.) braziliensis* and *L. (V.) peruviana*, and *L. (V.) guyanensis* and *L. (V.) panamensis*. However, genetic exchange will not occur between more distantly-related *Leishmania* species within a sand fly, when its affinities differ for those *Leishmania* species. Therefore, the potential for genetic exchange within reservoirs with subsequent hybrid formation should be considered.

## Mito-Nuclear Discordance

In addition to hybrid species, recent studies have reported the presence of *Leishmania* strains showing discordance between kDNA and nDNA at a relatively high rate; this represents the first report of mito-nuclear discordance in protozoan parasites (Kato et al., 2019b; Tabbabi et al., 2020). It is not known where and how such incompatibility occurs, or whether the mechanism is the same as for the hybrid formation of nDNA.

Mitochondrial and nuclear genomes co-exist in each cell; however, the rate of evolution of mitochondrial DNA (mtDNA) is more rapid than that of nDNA (Toews and Brelsford, 2012). Incompatibility between mtDNA and nDNA has been reported in various organisms including mammals, birds, reptiles, amphibians, fish, insects, and yeasts (Toews and Brelsford, 2012). It has also been reported in helminth parasites, between *Schistosoma turkestanicum* populations (Lawton et al., 2017), between *Taenia solium* lineages (Yanagida et al., 2014), and between *T. saginata* and *T. asiatica* (Yamane et al., 2012; Yamane et al., 2013; Sato et al., 2018). Mito-nuclear discordance is considered to result from various processes such as adaptive introgression of mtDNA, demographic disparities, sex-biased asymmetries, hybrid zone movement, an intracellular bacteria, *Wolbachia* infection in insects, and human actions (Toews and Brelsford, 2012). It is well known that mitochondria play essential roles in cellular energy production, cellular proliferation, and many other metabolic functions (McBride et al., 2006). Although mitochondria contain their own DNA independently, the interaction between mitochondrial and nuclear genomes is important for biological functions of the cell (McBride et al., 2006; Ali et al., 2019). Therefore, it is considered that exchange of kDNA possibly affects pathogenicity and transmission potential by sand flies of *Leishmania* protozoa, as suggested in a hybrid strain (Cortes et al., 2012). Further studies that involve isolating parasite strains with mito-nuclear discordance are expected to elucidate these issues and provide further insight into the mechanism of genetic exchange between *Leishmania* protozoa.

## Concluding Remarks

This review describes the genetic exchange that results in the establishment of hybrid strains and mito-nuclear discordance in *Leishmania* under natural conditions. Formation of a hybrid strain was suggested to increase the severity of disease when compared with parental species in an experimental animal model (Cortes et al., 2012). In addition, hybrid strains can increase the potential for sand fly transmission (Volf et al., 2007; Seblova et al., 2015). It is not yet well-established whether strains showing mito-nuclear discordance have increased pathogenicity or vector range. Isolation of strains with mito-nuclear discordance and further studies on the infection in animals and sand flies will be necessary to clarify these issues. Since mitochondria are organelles that are essential for cell energy supply, differentiation, and growth, (McBride et al., 2006), the genetic exchange resulting in mito-nuclear discordance could affect disease progression, as well as modify the potential for transmission by sand flies. Finally, the development of hybrids and strains with mito-nuclear discordance may have biological significance for parasite evolution and adaptation.

## Author Contributions

All authors listed have made a substantial, direct, and intellectual contribution to the work and approved it for publication.

## Funding

This study was financially supported by the Ministry of Education, Culture and Sports, Science and Technology (MEXT) of Japan (Grant No. 17H01685).

## Conflict of Interest

The authors declare that the research was conducted in the absence of any commercial or financial relationships that could be construed as a potential conflict of interest.

## References

[B1] Akopyants N. S. Kimblin N. Secundino N. Patrick R. Peters N. Lawyer P.. (2009). Demonstration of genetic exchange during cyclical development of *Leishmania* in the sand fly vector. Science 324, 265–268. 10.1126/science.1169464 19359589 PMC2729066

[B2] Ali A. T. Boehme L. Carbajosa G. Seitan V. C. Small K. S. Hodgkinson A. (2019). Nuclear genetic regulation of the human mitochondrial transcriptome. eLife 8, e41927. 10.7554/eLife.41927 30775970 PMC6420317

[B3] Asato Y. Oshiro M. Myint C. K. Yamamoto Y. Kato H. Marco J. D.. (2009). Phylogenic analysis of the genus *Leishmania* by cytochrome *b* gene sequencing. Exp. Parasitol. 121, 352–361. 10.1016/j.exppara.2008.12.013 19159626

[B4] Bañuls A. L. Guerrini F. Le Pont F. Barrera C. Espinel I. Guderian R.. (1997). Evidence for hybridization by multilocus enzyme electrophoresis and random amplified polymorphic DNA between *Leishmania braziliensis* and *Leishmania panamensis/guyanensis* in Ecuador. J. Eukaryot. Microbiol. 44, 408–411. 10.1111/j.1550-7408.1997.tb05716.x 9304809

[B5] Calvo-Álvarez E. Álvarez-Velilla R. Jiménez M. Molina R. Pérez-Pertejo Y. Balaña-Fouce R.. (2014). First evidence of intraclonal genetic exchange in trypanosomatids using two *Leishmania infantum* fluorescent transgenic clones. PLoS Negl. Trop. Dis. 8, e3075. 10.1371/journal.pntd.0003075 25188587 PMC4154677

[B6] Cortes S. Esteves C. Mauricio I. Maia C. Cristovão J. M. Miles M.. (2012). *In vitro* and *in vivo* behavior of sympatric *Leishmania (V.) braziliensis, L. (V.) peruviana and* their hybrids. Parasitology 139, 191–199. 10.1017/S0031182011001909 22054424

[B7] Cupolillo E. Grimaldi G. Momen H. (1994). A general classification of New World *Leishmania* using numerical zymotaxonomy. Am. J. Trop. Med. Hyg. 50, 296–311. 10.4269/ajtmh.1994.50.296 8147488

[B8] da Silva L. A. de Sousa C. dos S. da Grac G. C. Porrozzi R. Cupolillo E. (2010). Sequence analysis and PCR RFLP profiling of the *hsp70* gene as a valuable tool for identifying *Leishmania* species associated with human leishmaniasis in Brazil. Infect. Genet. Evol. 10, 77–83. 10.1016/j.meegid.2009.11.001 19913112

[B9] de Almeida M. E. Steurer F. J. Koru O. Herwaldt B. L. Pieniazek N. J. da Silva A. J. (2011). Identification of *Leishmania* spp. by molecular amplification and DNA sequencing analysis of a fragment of rRNA internal transcribed spacer 2. J. Clin. Microbiol. 49, 3143–3149. 10.1128/JCM.01177-11 21752983 PMC3165567

[B10] Delgado O. Cupolillo E. Bonfante-Garrido R. Silva S. Belfort E. Júnior G. G.. (1997). Cutaneous leishmaniasis in Venezuela caused by infection with a new hybrid between *Leishmania (Viannia) braziliensis* and *L. (V.) guyanensis* . Mem. Inst. Oswaldo Cruz 92, 581–582. 10.1590/s0074-02761997000500002 9566221

[B11] Dujardin J. C. Bañuls A. L. Llanos-Cuentas A. Alvarez E. DeDoncker S. Jacquet D.. (1995). Putative *Leishmania* hybrids in the Eastern Andean valley of Huanuco, Peru. Acta Trop. 59, 293–307. 10.1016/0001-706x(95)00094-u 8533665

[B13] Fraga J. Veland N. Montalvo A. M. Praet N. Boggild A. K. Valencia B. M.. (2012). Accurate and rapid species typing from cutaneous and mucocutaneous leishmaniasis lesions of the New World. Diagn. Microbiol. Infect. Dis. 74, 142–150. 10.1016/j.diagmicrobio.2012.06.010 22819605

[B12] Fraga J. Montalvo A. M. Maes L. Dujardin J. C. Van der Auwera G. (2013). *Hin*dII and *Sdu*I digests of heat-shock protein 70 PCR for *Leishmania* typing. Diagn. Microbiol. Infect. Dis. 77, 245–247. 10.1016/j.diagmicrobio.2013.07.023 24050933

[B14] Garcia L. Kindt A. Bermudez H. Llanos-Cuentas A. De Doncker S. Arevalo J.. (2004). Culture-independent species typing of neotropical *Leishmania* for clinical validation of a PCR-based assay targeting heat shock protein 70 genes. J. Clin. Microbiol. 42, 2294–2297. 10.1128/jcm.42.5.2294-2297.2004 15131217 PMC404633

[B16] Hashiguchi Y. Gomez E. L. Kato H. Martini L. R. Velez L. N. Uezato H. (2016). Diffuse and disseminated cutaneous leishmaniasis: clinical cases experienced in Ecuador and a brief review. Trop. Med. Health 44, 2. 10.1186/s41182-016-0002-0 27398061 PMC4934146

[B17] Hashiguchi Y. Velez L. N. Villegas N. V. Mimori T. Gomez E. A. L. Kato H. (2017). Leishmaniases in Ecuador: Comprehensive review and current status. Acta Trop. 166, 299–315. 10.1186/s41182-016-0002-0 27919688

[B15] Hashiguchi Y. Gomez E. A. L. Cáceres A. G. Velez L. N. Villegas N. V. Hashiguchi K.. (2018). Andean cutaneous leishmaniasis (Andean-CL, uta) in Peru and Ecuador: the causative *Leishmania* parasites and clinico-epidemiological features. Acta Trop. 177, 135–145. 10.1016/j.actatropica.2017.09.028 29017878

[B18] Kamhawi S. Ramalho-Ortigao M. Pham V. M. Kumar S. Lawyer P. G. Turco S. J.. (2004). A role for insect galectins in parasite survival. Cell 119, 329–341. 10.1016/j.cell.2004.10.009 15543683

[B20] Kato H. Caceres A. G. Mimori T. Ishimaru Y. Sayed A. S. Fujita M.. (2010). Use of FTA cards for direct sampling of patient’s lesions in the ecological study of cutaneous leishmaniasis. J. Clin. Microbiol. 48, 3661–3665. 10.1128/JCM.00498-10 20720027 PMC2953078

[B26] Kato H. Watanabe J. Mendoza Nieto I. Korenaga M. Hashiguchi Y. (2011). *Leishmania* species identification using FTA card sampling directly from patients’ cutaneous lesions in the state of Lara, Venezuela. Trans. R. Soc Trop. Med. Hyg. 105, 561–567. 10.1016/j.trstmh.2011.05.009 21907375

[B22] Kato H. Calvopiña M. Criollo H. Hashiguchi Y. (2013). First human cases of *Leishmania (Viannia) naiffi* infection in Ecuador and identification of its suspected vector species. Acta Trop. 128, 710–713. 10.1016/j.actatropica.2013.09.001 24044975

[B24] Kato H. Gomez E. A. Martini-Robles L. Muzzio J. Velez L. Calvopiña M.. (2016a). Geographic distribution of *Leishmania* species in Ecuador based on the cytochrome *b* gene sequence analysis. PLoS Negl. Trop. Dis. 10, e0004844. 10.1371/journal.pntd.0004844 27410039 PMC4943627

[B19] Kato H. Bone A. E. Mimori T. Hashiguchi K. Shiguango G. F. Gonzales S. V.. (2016b). First human cases of *Leishmania (Viannia) lainsoni* infection and a search for the vector sand flies in Ecuador. PLoS Negl. Trop. Dis. 10, e0004728. 10.1371/journal.pntd.0004728 27191391 PMC4871579

[B23] Kato H. Cáceres A. G. Hashiguchi Y. (2016c). First evidence of a hybrid of *Leishmania (Viannia) braziliensis/L. (V.) peruviana* DNA detected from the *Phlebotomine* sand fly *Lutzomyia tejadai* in Peru. PLoS Negl. Trop. Dis. 10, e0004336. 10.1371/journal.pntd.0004336 26735142 PMC4703407

[B21] Kato H. Caceres A. G. Seki C. Silupu Garcia C. R. Holguin Mauricci C. Castro Martinez S. C.. (2019a). Further insight into the geographic distribution of *Leishmania* species in Peru by cytochrome *b* and mannose phosphate isomerase gene analyses. PLoS Negl. Trop. Dis. 13, e0007496. 10.1371/journal.pntd.0007496 31220120 PMC6605678

[B25] Kato H. Gomez E. A. Seki C. Furumoto H. Martini-Robles L. Muzzio J.. (2019b). PCR-RFLP analyses of *Leishmania* species causing cutaneous and mucocutaneous leishmaniasis revealed distribution of genetically complex strains with hybrid and mito-nuclear discordance in Ecuador. PLoS Negl. Trop. Dis. 13, e0007403. 10.1371/journal.pntd.0007403 31059516 PMC6522058

[B27] Khanra S. Datta S. Mondal D. Saha P. Bandopadhyay S. K. Roy S.. (2012). RFLPs of ITS, ITS1 and hsp70 amplicons and sequencing of ITS1 of recent clinical isolates of Kala-azar from India and Bangladesh confirms the association of *L. tropica* with the disease. Acta Trop. 124, 229–234. 10.1016/j.actatropica.2012.08.017 22960646

[B28] Koarashi Y. Caceres A. G. Zuniga S. M. F. Palacios F. E. E. Celis T. A. Abanto A. J. L.. (2016). Identification of causative *Leishmania* species in Giemsa-stained smears prepared from patients with cutaneous leishmaniasis in Peru using PCR-RFLP. Acta Trop. 158, 83–87. 10.1016/j.actatropica.2016.02.024 26943992

[B29] Lawton S. P. Bowen L. I. Emery A. M. Majoros G. (2017). Signatures of mito-nuclear discordance in *Schistosoma turkestanicum* indicate a complex evolutionary history of emergence in Europe. Parasitol 144, 1752–1762. 10.1017/S0031182017000920 28747240

[B30] Leelayoova S. Siripattanapipong S. Hitakarun A. Kato H. Tan-ariya P. Siriyasatien P.. (2013). Multilocus characterization and phylogenetic analysis of *Leishmania siamensis* isolated from autochthonous visceral leishmaniasis cases, southern Thailand. BMC Microbiol. 13:60. 10.1186/1471-2180-13-60 23506297 PMC3724499

[B31] Louradour I. Ferreira T. R. Ghosh K. Shaik J. Sacks D. (2020). *In vitro* generation of *Leishmania* hybrids. Cell Rep. 31, 107507. 10.1016/j.celrep.2020.03.071 32294444

[B32] Luyo-Acero G. E. Uezato H. Oshiro M. Takei K. Kariya K. Katakura K.. (2004). Sequence variation of the cytochrome *b* gene of various human infecting members of the genus *Leishmania* and their phylogeny. Parasitol 128, 483–491. 10.1017/s0031182004004792 15180316

[B33] McBride H. M. Neuspiel M. Wasiak S. (2006). Mitochondria: more than just a powerhouse. Curr. Biol. 16, R551–R560. 10.1016/j.cub.2006.06.054 16860735

[B34] Montalvo A. M. Fraga J. Maes I. Dujardin J. C. Van der Auwera G. (2012). Three new sensitive and specific heat shock protein 70 PCRs for global *Leishmania* species identification. Eur. J. Clin. Microbiol. Infect. Dis. 31, 1453–1461. 10.1007/s10096-011-1463-z 22083340

[B35] Mouttaki T. Morales-Yuste M. Merino-Espinosa G. Chiheb S. Fellah H. Martin-Sanchez J.. (2014). Molecular diagnosis of cutaneous leishmaniasis and identification of the causative *Leishmania* species in Morocco by using three PCR-based assays. Parasitol. Vectors 7:420. 10.1186/1756-3305-7-420 PMC4161773 25189460

[B36] Odiwuor S. De Doncker S. Maes I. Dujardin J. C. Van der Auwera G. (2011). Natural *Leishmania donovani/Leishmania aethiopica* hybrids identified from Ethiopia. Infect. Genet. Evol. 11, 2113–2118. 10.1016/j.meegid.2011.04.026 21558020

[B37] Paranaiba L. F. Pinheiro L. J. Torrecilhas A. C. Macedo D. H. Menezes-Neto A. Tafuri W. L.. (2017). *Leishmania enriettii* (Muniz & Medin ): A highly diverse parasite is here to stay. PLoS Pathog. 13, e1006303. 10.1371/journal.ppat.1006303 PMC5444841 28542526

[B38] Ravel C. Cortes S. Pratlong F. Morio F. Dedet J. P. Campino L. (2006). First report of genetic hybrids between two very divergent *Leishmania* species: *Leishmania infantum* and *Leishmania major* . Int. J. Parasitol. 36, 1383–1388. 10.1016/j.ijpara.2006.06.019 16930606

[B39] Rioux J. A. Lanotte G. Serres E. Pratlong F. Bastien P. Perieres J. (1990). Taxonomy of *Leishmania.* Use of isoenzymes. Suggestions for a new classification. Ann. Parasitol. Hum. Comp. 65, 111–125. 10.1051/parasite/1990653111 2080829

[B40] Rogers M. B. Downing T. Smith B. A. Imamura H. Sanders M. Svobodova M.. (2014). Genomic confirmation of hybridisation and recent inbreeding in a vector-isolated *Leishmania* population. PLoS Genet. 10, e1004092. 10.1371/journal.pgen.1004092 24453988 PMC3894156

[B41] Rotureau B. Ravel C. Couppie P. Pratlong F. Nacher M. Dedet J. P.. (2006). Use of PCR-restriction fragment length polymorphism analysis to identify the main new world *Leishmania* species and analyze their taxonomic properties and polymorphism by application of the assay to clinical samples. J. Clin. Microbiol. 44, 459–467. 10.1128/JCM.44.2.459-467.2006 16455899 PMC1392689

[B42] Ruiz-Postigo J. A. Grouta L. Jaina S. (2020). Global leishmaniasis surveillance 2017–2018, and first report on 5 additional indicators. World Health Organ. Wkly. Epidemiological Rec. 25, 265–280.

[B43] Sadlova J. Yeo M. Seblova V. Lewis M. D. Mauricio I. Volf P.. (2011). Visualisation of *Leishmania donovani* fluorescent hybrids during early stage development in the sand fly vector. PLoS One 6, e19851. 10.1371/journal.pone.0019851 21637755 PMC3103508

[B44] Sato M. O. Sato M. Yanagida T. Waikagul J. Pongvongsa T. Sako Y.. (2018). *Taenia solium, Taenia saginata, Taenia asiatica*, their hybrids and other helminthic infections occurring in a neglected tropical diseases’ highly endemic area in Lao PDR. PLoS Negl. Trop. Dis. 12, e0006260. 10.1371/journal.pntd.0006260 29420601 PMC5821399

[B45] Seblova V. Myskova J. Hlavacova J. Votypka J. Antoniou M. Volf P. (2015). Natural hybrid of *Leishmania infantum/L. donovani:* development in *Phlebotomus tobbi*, *P. perniciosus* and *Lutzomyia longipalpis* and comparison with non-hybrid strains differing in tissue tropism. Parasitol. Vectors 8, 605. 10.1186/s13071-015-1217-3 PMC4660806 26608249

[B46] Simpson L. (1986). Kinetoplast DNA in trypanosomid flagellates. Int. Rev. Cytol. 99, 119–179. 10.1016/s0074-7696(08)61426-6 3082787

[B47] Spanakos G. Piperaki E. T. Menounos P. G. Tegos N. Flemetakis A. Vakalis N. C. (2008). Detection and species identification of Old World *Leishmania* in clinical samples using a PCR-based method. Trans. R. Soc Trop. Med. Hyg. 102, 46–53. 10.1016/j.trstmh.2007.05.019 17669452

[B48] Tabbabi A. Cáceres A. G. Bustamante Chauca T. P. Seki C. Choochartpong Y. Mizushima D.. (2020). Nuclear and kinetoplast DNA analyses reveal genetically complex *Leishmania* strains with hybrid and mito-nuclear discordance in Peru. PLoS Negl. Trop. Dis. 14, e0008797. 10.1371/journal.pntd.0008797 33075058 PMC7595639

[B49] Talmi-Frank D. Nasereddin A. Schnur L. F. Schonian G. Toz S. O. Jaffe C. L.. (2010). Detection and identification of Old World *Leishmania* by high resolution melt analysis. PLoS Negl. Trop. Dis. 4, e581. 10.1371/journal.pntd.0000581 20069036 PMC2797090

[B50] Toews D. P. Brelsford A. (2012). The biogeography of mitochondrial and nuclear discordance in animals. Mol. Ecol. 21, 3907–3930. 10.1111/j.1365-294X.2012.05664.x 22738314

[B51] Van den Broeck F. Savill N. J. Imamura H. Sanders M. Maes I. Cooper S.. (2020). Ecological divergence and hybridization of Neotropical *Leishmania* parasites. Proc. Natl. Acad. Sci. U. S. A. 117, 25159–25168. 10.1073/pnas.1920136117 32958676 PMC7547230

[B52] Volf P. Benkova I. Myskova J. Sadlova J. Campino L. Ravel C. (2007). Increased transmission potential of *Leishmania major/Leishmania infantum* hybrids. Int. J. Parasitol. 37, 589–593. 10.1016/j.ijpara.2007.02.002 17376453 PMC2839924

[B53] Yamane K. Suzuki Y. Tachi E. Li T. Chen X. Nakao M.. (2012). Recent hybridization between *Taenia asiatica* and *Taenia saginata* . Parasitol. Int. 61, 351–355. 10.1016/j.parint.2012.01.005 22301089

[B54] Yamane K. Yanagida T. Li T. Chen X. Dekumyoy P. Waikagul J.. (2013). Genotypic relationships between *Taenia saginata*, *Taenia asiatica* and their hybrids. Parasitology 140, 1595–1601. 10.1017/S0031182013001273 24112449

[B55] Yanagida T. Carod J. F. Sako Y. Nakao M. Hoberg E. P. Ito A. (2014). Genetics of the pig tapeworm in Madagascar reveal a history of human dispersal and colonization. PLoS One 9, e109002. 10.1371/journal.pone.0109002 25329310 PMC4198324

